# Urinary extracellular vesicle dynamics in Parkinson's disease patients with urinary dysfunction

**DOI:** 10.3389/fneur.2023.1250832

**Published:** 2023-11-17

**Authors:** Santanu Roy, Namita N. Kashyap, Abigail Sheldon Anchan, Dhiren Punja, Dushyanth Babu Jasti, Dinesh Upadhya

**Affiliations:** ^1^Centre for Molecular Neurosciences, Kasturba Medical College, Manipal Academy of Higher Education, Manipal, Karnataka, India; ^2^Department of Physiology, Kasturba Medical College, Manipal Academy of Higher Education, Manipal, Karnataka, India; ^3^Department of Microbiology, Kasturba Medical College, Manipal Academy of Higher Education, Manipal, Karnataka, India; ^4^Department of Neurology, Kasturba Medical College, Manipal Academy of Higher Education, Manipal, Karnataka, India

**Keywords:** extracellular vesicles, Parkinson's disease, motor symptoms, urinary dysfunctions, neurodegenerative disease

## Abstract

Parkinson's disease (PD) presents with severe motor manifestations and a plethora of non-motor symptoms. Urinary dysfunctions are one of the most common non-motor symptoms of PD patients responsible for reduced quality of life. Urinary extracellular vesicles (EVs) are mostly considered to originate from the cells in the urogenital tract. In this study, we have performed urinary EV analysis in 29 PD cases with varied severity of urinary dysfunction and correlated it with the EV dynamics in 29 age-matched controls. In the studied cases, apart from urinary dysfunction, symptoms of depression, anxiety, cognitive dysfunction, sleep, and wakefulness were observed in >75% of the cases. No significant difference in urinary EV size, concentration and urinary EV protein concentration was observed between PD cases with urinary dysfunction and controls. However, a significant positive association was observed between urinary EV concentration and motor scores (*p* = 0.042), while no association was observed between urinary EV concentration and urinary dysfunction scores. Chronic stress induced by motor symptoms could be one of the reasons for excessive EV production in PD patients with urinary dysfunctions. Large-scale studies on the association of urinary EV dynamics with motor and non-motor symptoms may provide additional information on urinary dysfunction in PD.

## Introduction

Parkinson's disease (PD) presents with basic motor manifestations of bradykinesia, unequal tremor, and rigidity. Apart from motor symptoms, a plethora of non-motor symptoms (NMS) is well-associated with PD. The NMS are frequent, may be observed far before the actual onset of motor symptoms, and may correlate to a variable extent with the severity of motor symptoms (1, 2). The NMS could be observed throughout the course of PD and is considered the most important determinant of quality of life for PD patients. They are also responsible for the increased cost of care and hospitalization in the late stages of PD (3, 4). Urinary dysfunctions are one of the most common non-motor symptoms of Parkinson's disease patients responsible for reduced quality of life (5, 6). A recent systematic review and meta-analysis revealed urinary dysfunction in >60% of PD cases (6).

The term “extracellular vesicles” (EVs) refers to particles that are naturally released from cells, are delimited by a lipid bilayer, and cannot replicate independently (7). EVs are known to be released from all cell types. EVs contain proteins, lipids, mRNAs, miRNAs, and several other biomolecules depending on the parental cell source (8, 9). Since urine is available in plenty non-invasively, it is an easy source for exploring disease associations. Urinary extracellular vesicles are mostly considered to originate from the cells in the urogenital tract. Thus, urinary EV dynamics may be altered with the pathology of kidneys, bladder, and male or female urogenital tracts (10, 11). As PD is well-associated with urinary dysfunction, identifying the urinary EV dynamics in PD cases with urinary dysfunction and comparing it with age-matched controls could provide new insights into the pathophysiology of this NMS. In this study, we have performed EV analysis in 29 PD cases with varied severity of urinary dysfunction and correlated it with the EV dynamics in 29 age-matched controls.

## Materials and methods

### Study subjects and their clinical diagnosis

This study was a prospective cross-sectional single-center study performed following approval from the Kasturba Medical College Institutional Ethics Committee (# IEC 43/2021). Parkinson's-plus syndromes like multiple system atrophy, progressive supranuclear palsy, corticobasal degeneration and secondary Parkinsonism due to multiple causes were excluded. Following informed consent, 29 patients were recruited for PD cases and the demographic data were collected. Of the 29 cases, 26 cases examined were Hoehn and Yahr stage 2 with tremor rigidity affecting bilaterally, and walking/gait difficulties but without a history of falls. Only three cases were stage 1 with unilateral motor involvement. All the PD cases except two naive cases were on levodopa and carbidopa therapy thrice daily. All PD patients were assessed only in the “on” period for logistic reasons. On and off state refers to the motor fluctuation with the gradual decrease in the levodopa effect. On period refers to when motor symptoms are well-controlled. The off period refers to when the motor symptoms return.

### Identifying motor symptoms

Along with bradykinesia, the presence of rigidity, 4–6 Hz resting tremor, or postural instability along with three supportive features were considered for PD diagnosis. The Movement Disorders Society—Unified Parkinson's Disease Rating Scale (UPDRS) characterizes the extent and burden of the disease and defines the disease course. Part 3 of the UPDRS is for motor examination and the specific disability is marked on a 0–4 scale which requires about 10 min for completion.

### Identifying non-motor symptoms

Non-motor rating scale was used to assess the extent of various motor and NMSs symptoms. The NMS section comprises 52 items gathered into 13 distinct domains: five items in depression, six in cognition, three in apathy, four in psychosis, four items in anxiety, four in impulse control and related disorders, three in urinary, two in orthostatic hypotension, two in sexual, six in sleep and wakefulness, four in gastrointestinal, four in pain and five in others. Scoring was done for frequency (0–4, 0 being never to 4 being most of the time) and severity (0 to 4, zero being not present to 4 being severe). These two values were multiplied to get the total score. The maximum possible score is 832 points.

### Urine collection

From each PD patient, a urine sample was collected in a 20 ml sterile container labeled with the sample number. For comparing the dynamics of extracellular vesicles, urine was collected from 29 age-matched control, healthy cases.

### Isolation of urinary extracellular vesicles

Twenty ml of a urine sample from the sterile container was transferred to a 50 ml Falcon tube. The first centrifugation was done at 2,500 rcf for 30 min at 4°C. Pellet was discarded and to the supernatant, an equal quantity of 12% 2x polyethylene glycol was added and incubated at 4°C for 1 h. After 1 h, centrifugation was carried out at 3,300 rcf for 1 h at 4°C. The pellet was resuspended in sterile 1 ml PBS and an equal quantity of 5% 2x polyethylene glycol was added and incubated at 4°C for 1 h. After 1 h, centrifugation was carried out at 3,300 rcf for 1 h at 4°C. The supernatant was discarded and the pellet was collected.

### Evaluation of size distribution and concentration of EVs

Purified EVs were characterized according to Minimum Information for the Study of Extracellular Vesicles (MISEV) 2018 guidelines (7). The size distribution and the concentration of urinary EVs were determined by NanoSight LM10 (Malvern Instruments, UK). Detailed procedures for sample analysis were provided in our earlier report (12).

### Morphology of EVs

The morphology of the urinary EVs was evaluated using transmission electron microscopy by applying a few drops of isolated EVs to 300 mesh carbon-coated grids at room temperature. These grids were stained with 0.5% uranyl acetate, and air dried for 10 min at room temperature. The images were collected using JEM 2100, a multipurpose 120 KV analytical transmission microscope.

### Evaluation of molecular marker expression in the urinary EVs

The molecular indicators for EVs, such as positive markers TSG 101 and CD 63 proteins, and negative marker GRP 94, were evaluated through western blotting. As a positive control for TSG101, CD63, and GRP94, human embryonic kidney (HEK293) cells were used. Briefly, following the lysis of EV and cell samples in RIPA buffer, total proteins in the isolated EVs were estimated using the BCA method. Proteins were separated through SDS-PAGE and transferred to the PVDF membrane. The presence of TSG101, CD63, and GRP94 was detected using rabbit polyclonal antibodies PA 531260, SAB 4301607, and SAB 2101094 (all from Invitrogen), respectively with appropriate secondary antibodies. The initial protein loading amount was confirmed with monoclonal β-actin (MA 1140, Invitrogen).

### Statistical analysis

Statistical analysis was performed using GraphPad Prism. Results were expressed as either Mean ± SEM or percentage. The student unpaired *t*-test was done to compare results between the two groups. Linear regression analysis was performed to find the association between different parameters.

## Results

The study population included 29 idiopathic Parkinson's disease cases and 29 age-matched controls. Individual patient characteristics are provided in Table 1. There was a total of 12 female PD patients. The mean age of 29 controls was 61.2 ± 1.98 years while the mean age of 29 PD cases was 60.2 ± 1.7 years with no significant difference between the two groups. PD cases were confirmed based on the motor examination as per UPDRS part 3.

**Table 1:** Demonstrates individual patient characteristics.

**Patient ID**	**Gender**	**Age (years)**	**Total UPDRS score**	**Total NMS score**	**Duration of PD (years)**
1	F	50	9	169	8
2	M	66	32	38	4
3	M	51	13	125	3
4	M	71	28	77	1.5
5	F	60	34	202	10
6	F	49	10	166	3
7	M	61	24	199	2
8	M	77	50	234	2
9	M	67	18	268	8
10	M	70	18	148	4
11	M	56	28	24	3
12	F	61	29	410	4
13	F	48	28	16	2
14	M	47	3	110	3
15	M	64	14	301	2
16	M	67	18	228	2
17	F	71	49	372	6
18	M	70	63	78	6
19	F	55	46	173	4
20	F	47	16	49	3
21	F	59	10	49	1
22	M	60	33	30	2
23	F	53	13	136	3
24	F	69	22	57	0.8
25	M	68	19	14	3
26	M	47	18	209	2
27	F	69	19	303	3.5
28	M	67	31	112	2
29	M	48	17	275	3

### Non-motor symptoms of Parkinson's disease patients

The NMS was evaluated for all 29 PD cases. Symptoms such as depression, anxiety, cognitive dysfunction, urinary dysfunction, sleep, and wakefulness were observed in >75% of the cases while apathy, gastrointestinal issues, and pain were demonstrated in >50% of the cases. Symptoms of psychosis, impulse control-related disorders, and orthostatic hypotension were observed in <50% of the cases.

### The severity of motor and non-motor symptoms

**Figure 1:**
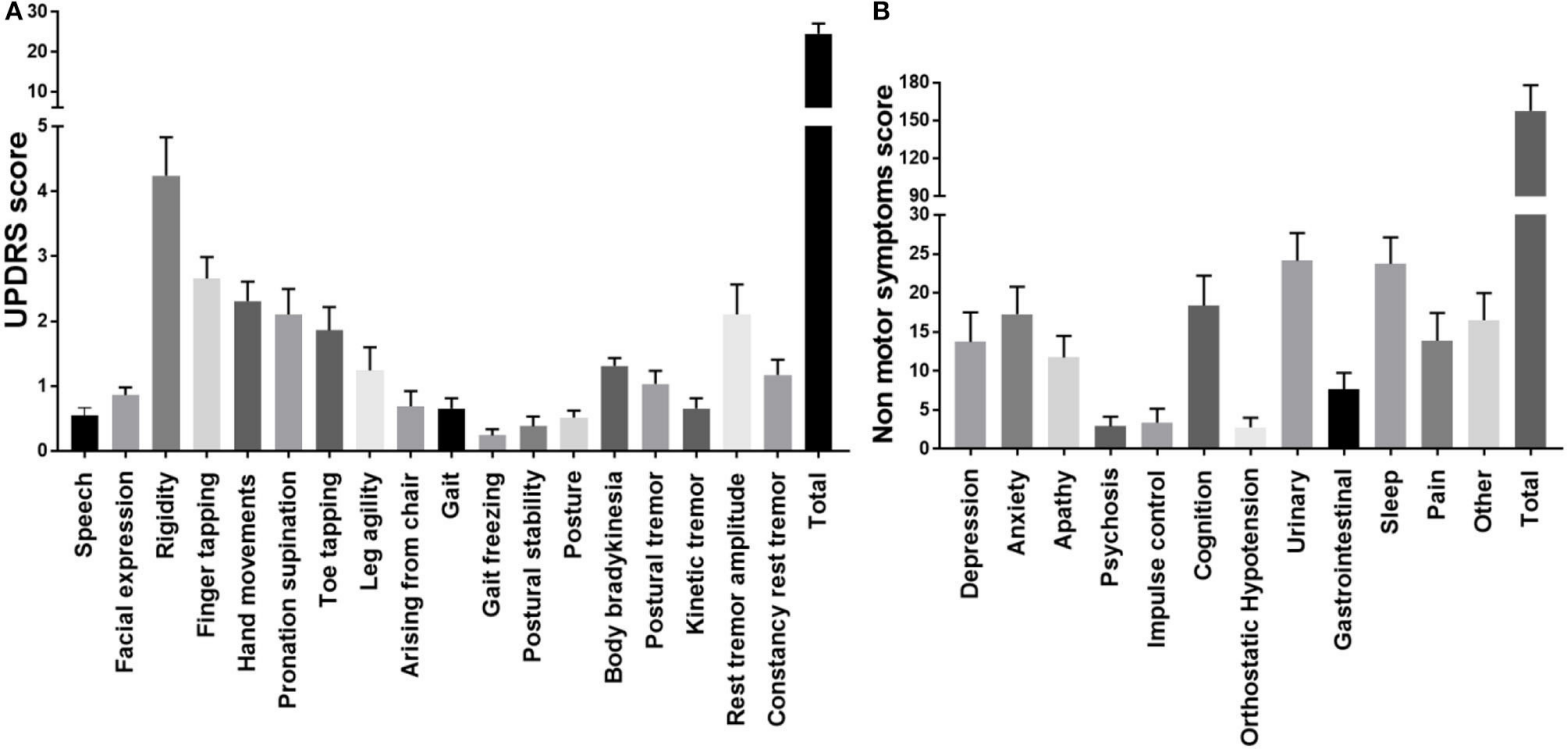
The severity of motor and non-motor symptoms in Parkinson's disease patients. **(A)** Severity of motor symptoms in PD cases. **(B)** Severity of non-motor symptoms in PD cases.

The severity of various motor symptoms is demonstrated in Figure 1A. The mean UPDRS part 3 score for 29 PD cases was 24.6 ± 2.6. The severity of muscle rigidity, finger tapping, hand movements, pronation supination, toe-tapping, and rest tremor amplitudes was highly demonstrated compared to other symptoms. Speech disturbance and mask facies were present in several cases but in lower severity. Gait and posture disturbance was found among a lesser number of cases.

The severity of NMS is demonstrated in Figure 1B. The mean NMS score was 161.66. The most severe NMS observed was urinary symptoms (24.14 ± 3.542) which include urinary urgency (urgent need to empty bladder); urinary frequency (had to empty the bladder more than every 2 h) and nocturia (had to empty the bladder more than twice overnight). The next most severe NMS was observed in the sleep domain (23.72 ± 3.392). These include insomnia sleep behavior, sleeping during waking hours, restlessness, periodic limb movements, waking due to snoring, and gasping. As seen in the graph below severity of anxiety, cognitive difficulties, and others (physical fatigue, mental fatigue, unintentional weight loss, impaired olfaction, and excessive sweating) were also high.

### Evaluation of urinary extracellular vesicles

**Figure 2:**
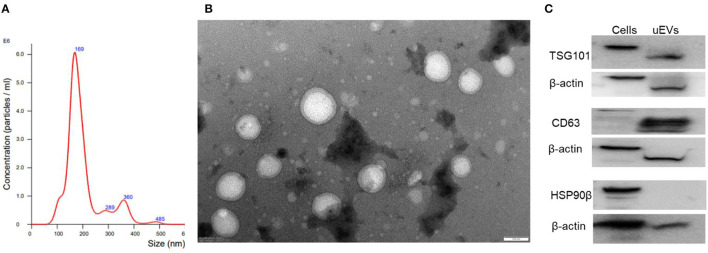
Characterization of isolated urinary EVs. **(A)** Nanoparticle tracking analysis of urinary EVs. **(B)** Ultrastructural evaluation of EVs using transmission electron microscopy. **(C)** Molecular markers of EVs TSG101, CD 63, and GRP 94 in isolated urinary EVs.

As mentioned in the methodology, urine samples were collected from all 29 PD cases and 29 age-matched controls. Isolated urinary extracellular vesicles (uEVs) were analyzed for the concentration and size distribution of EVs (Figure 2A), the ultrastructure of EVs (Figure 2B) and the molecular markers of the EVs (Figure 2C). As per the guidelines of MISEV 2018, evaluation of particle number and size (through nanoparticle tracking analysis, etc.), the ultrastructure of the isolated particle (using transmission electron microscopy, etc.) and three molecular markers (one cytosolic EV marker such as TSG101, one transmembrane EV marker such as CD63 and a negative EV marker such as GRP 94) is essential to confirm the identity of the isolated particles as EVs (7).

Detailed analysis of urinary EVs demonstrated a mean size of 211.4 ± 6.1 in controls and 202.5 ± 4.6 for PD cases without any significant difference between the groups. Ultrastructural evaluation of the EVs using transmission electron microscopy identified abundant ~200 nm-sized round or oval-shaped particles (Figure 2B). Evaluating molecular markers through western blotting of the isolated particles demonstrated the positive staining for TSG 101 and CD63, while negative for GRP 94 (Figure 2C). The positive control used confirmed the functionality of the GRP 94 antibody while β-actin immunostaining confirmed comparable loading of proteins for all the markers. These details confirmed that isolated particles from urine were indeed extracellular vesicles.

### Urinary EV size, concentration, and protein concentration in controls and PD cases with urinary dysfunction

**Figure 3:**
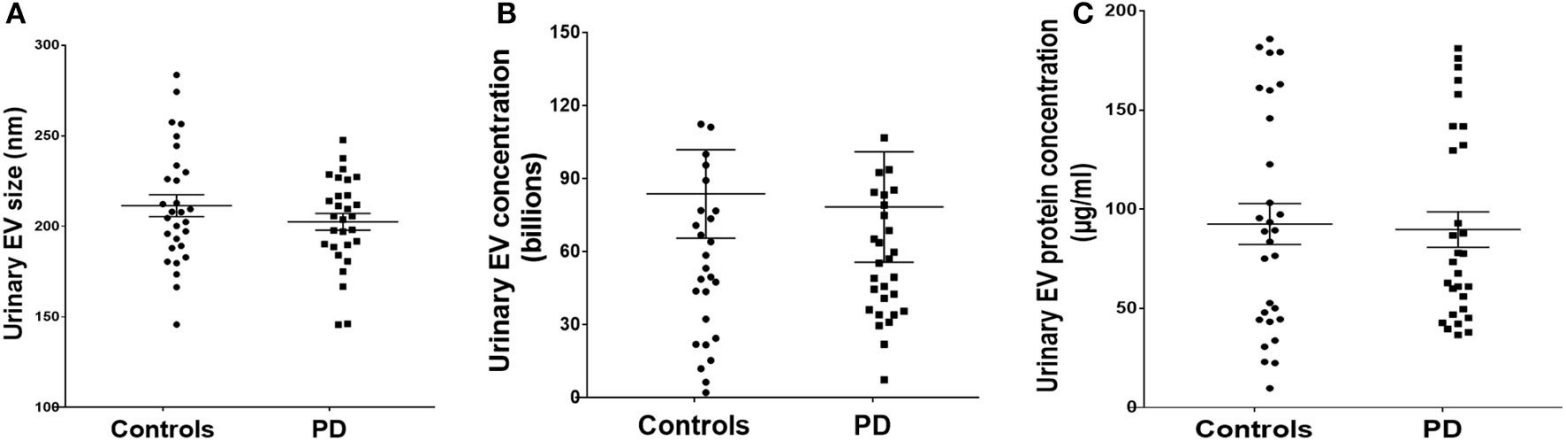
Urinary EV dynamics of PD and controls. **(A)** Demonstrates EV size, **(B)** demonstrates EV concentration, and **(C)** demonstrates EV protein concentration in control and PD cases with urinary dysfunction.

Detailed analysis of EV size demonstrated a mean size of 211.4 ± 6.1 in controls and 202.5 ± 4.6 for PD cases without any significant difference between the groups (Figure 3A). Analysis of urinary EV concentration demonstrated 83.74 ± 18.14 billion in controls and 78.39 ± 22.71 billion in PD patients demonstrating no significant difference between the groups (Figure 3B). Evaluation of urinary EV protein concentration demonstrated 92.56 ± 10.34 μg/ml for controls and 89.78 ± 8.95 μg/ml in PD patients demonstrating no significant difference between the groups (Figure 3C).

### Relationships of urinary EV size, concentration, and EV protein concentration with the severity of motor and non-motor symptoms of PD patients

**Figure 4:**
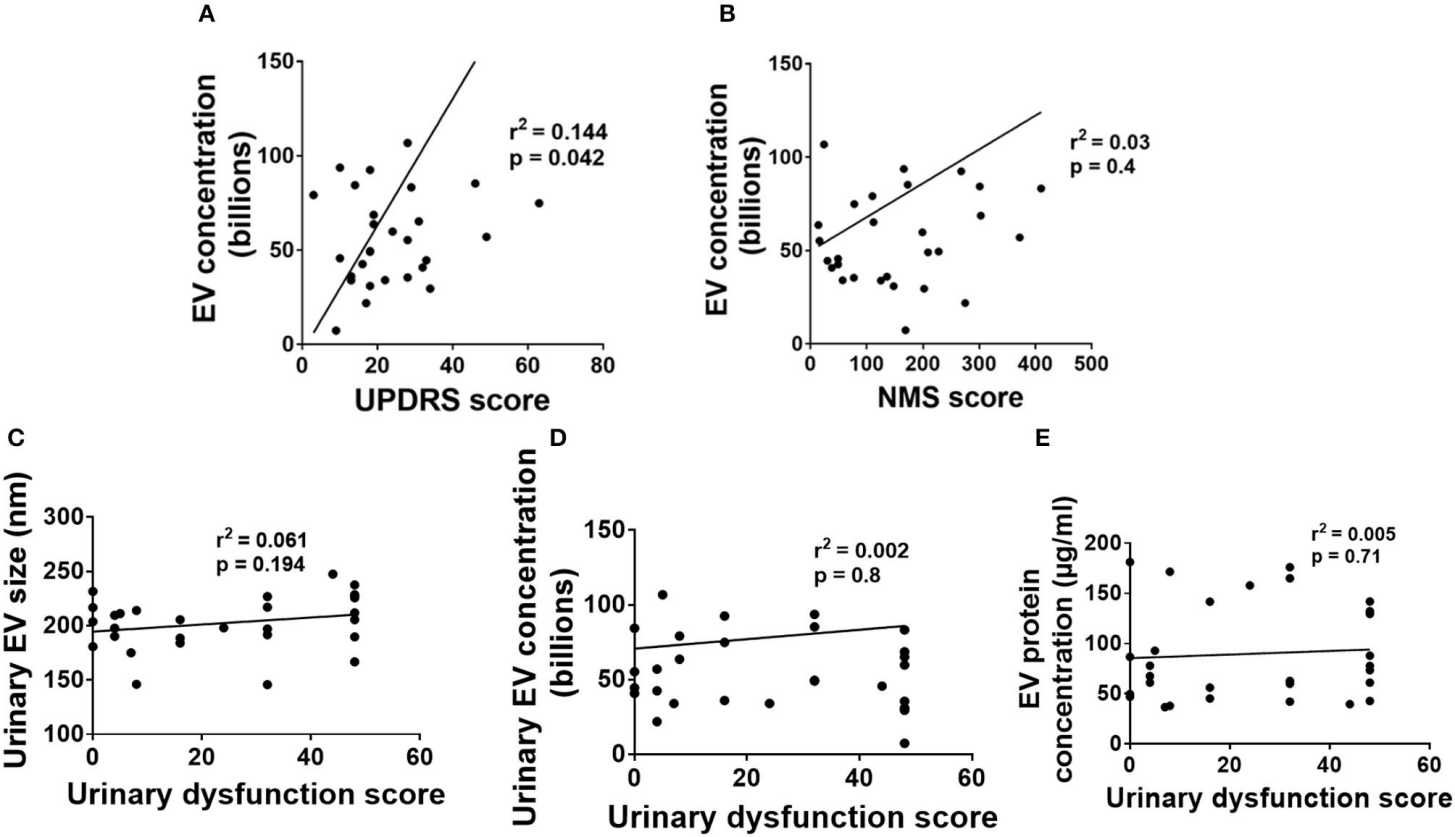
Association of urinary EV dynamics with PD symptoms. **(A)** Urinary EV concentration vs. UPDRS score. **(B)** Urinary EV concentration vs. nonmotor symptom score. **(C)** Urinary EV size with urinary dysfunction scores. **(D)** Urinary EV concentration with urinary dysfunction scores. **(E)** Urinary EV protein concentration with urinary dysfunction scores.

Linear regression analysis of urinary EV concentration separately with motor and NMS scores demonstrated a positive correlation (with motor symptoms *r*^2^ = 0.144, *p* = 0.042, Figure 4A) while no correlations with NMS (*r*^2^ = 0.03, *p* = 0.4, Figure 4B). The association of the severity of motor symptoms with urinary EV concentration is a very surprising finding and requires large-scale studies for the essential conclusion. However, urinary EV size and EV protein concentrations demonstrated no significant association with motor and NMS (data not shown).

### Relationships of urinary EV size, concentration, and EV protein concentration with urinary dysfunctions in PD patients

We have performed linear regression analysis for urinary EV data with urinary dysfunction scores. Linear regression analysis of urinary EV size with urinary dysfunction scores demonstrated no correlations between them (*p* > 0.05, Figure 4C). Also, linear regression analysis of urinary EV concentration with urinary dysfunction scores demonstrated no correlations between them (*p* > 0.05, Figure 4D). Further, linear regression analysis of urinary EV protein concentration with urinary dysfunction scores also demonstrated no correlations between them (*p* > 0.05, Figure 4E).

## Discussion

The high severity of motor symptoms is a well-established reality in PD. As per UPDRS part 3, the clinical criteria for diagnosis of PD requires the presence of bradykinesia and 1 of the following features: rigidity, 4–6 Hz resting tremor, or postural instability, in addition, to three supportive features. Varied extents of NMS in PD were demonstrated in different studies (13–16). Our results suggest that all the PD patients demonstrated different NMS with varying severity although urinary symptoms as the most severe NMS followed by sleep disturbance.

Urinary extracellular vehicles provide a great opportunity to non-invasively understand certain cellular pathologies associated with various disorders. Since Parkinson's disease is associated with severe motor and non-motor symptoms including urinary tract dysfunction, we used this opportunity to understand the urinary EV dynamics in PD. It is assumed that urinary dysfunction in PD is caused by neurogenic dysfunction and not by urogenital dysfunction. Normal micturition requires active functioning of the bladder and urethral sphincters, which are controlled by the central nervous system through coordinated network activity of the sympathetic and parasympathetic system with the somatic nervous system to maintain urinary continence (17). Neurodegeneration in the autonomic nervous system and higher micturition centers could be responsible for neurogenic lower urinary tract dysfunction in PD. Since urinary extracellular vesicles are mostly considered to originate from the cells in the urogenital tract, it is reasonable that no significant difference in urinary EV size, concentration, and urinary EV protein concentration was observed between PD cases with urinary dysfunction and controls.

Also, urinary dysfunction in PD is well-associated with the degeneration of nigrostriatal dopaminergic neurons in early and untreated patients (18). Exploring basic features such as size, number, and total protein content of urinary EVs could be useful for identifying unestablished links between motor and non-motor symptoms with urinary dysfunctions. While some specific molecular biomarker studies have been performed earlier in urinary EVs from PD cases (19), the available literature does not demonstrate the implications of motor and non-motor symptoms of PD and its medications such as levodopa and carbidopa on EV secretions in urinary dysfunctions. Although the sample size is small, our study observed a significant positive correlation between motor symptoms and urinary EV concentration while no definitive association was observed with non-motor symptoms, including urinary dysfunction. We believe stress induced by motor symptoms could be a possible reason for excessive EV production in PD patients as stress is an inducer of EVs (20).

Limitations of the study include a relatively small sample size and patients were assessed only in the “on” period for logistic reasons. Future studies on a large population of PD cases with urinary dysfunction could provide a definitive link between motor symptoms and urinary EV concentration. Also, implications of the duration of PD and severity of motor and non-motor symptoms on the urinary EV concentration could be extensively evaluated. Also, future studies on the molecular composition of urinary EVs may help to delineate causal factors for urinary dysfunction such as intrinsic lower urinary tract factors vs. PD-dependent pathology.

## Data availability statement

The original contributions presented in the study are included in the article/supplementary material, further inquiries can be directed to the corresponding author.

## Ethics statement

The studies involving humans were approved by Kasturba Medical College Institutional Ethics Committee. The studies were conducted in accordance with the local legislation and institutional requirements. The participants provided their written informed consent to participate in this study.

## Author contributions

SR: Conceptualization, Formal analysis, Writing—original draft, Writing—review & editing. NK: Writing—review & editing, Methodology. AA: Writing—review & editing, Methodology. DP: Conceptualization, Methodology, Writing—review & editing, DJ: Conceptualization, Methodology, Writing—review & editing, DU: Conceptualization, Funding, Methodology, Formal analysis, Writing—original draft, Writing—review & editing.
